# Establishment of High-Throughput Screening Protocol Based on Isomerase Using *Geobacillus* sp. L-Rhamnose Isomerase

**DOI:** 10.4014/jmb.2507.07026

**Published:** 2025-08-26

**Authors:** Na Kyeong Koo, Sol Min Han, Seong-Bo Kim, Seung-Ho Baek, Hyun June Park

**Affiliations:** 1 Department of Bio-Health Convergence, Duksung Women’s University, Seoul 01369, Republic of Korea; 2 Department of Biotechnology, Duksung Women’s University, Seoul 01369, Republic of Korea; 3 Bio-Living Engineering Major, Yonsei University, Seoul 03722, Republic of Korea; 4 Center for Bio-based Chemistry, Korea Research Institute of Chemical Technology (KRICT), Ulsan 44429, Republic of Korea

**Keywords:** High-throughput screening, Isomerization, L-Rhamnose isomerase, Seliwanoff's reaction

## Abstract

Directed evolution is a powerful tool in protein engineering that generates diverse variant libraries to enhance enzyme functionalities. However, the identification of desirable variants from large mutant libraries requires an efficient high-throughput screening (HTS) technique. In this study, we established a robust HTS protocol for selecting high-activity isomerase variants, specifically using L-rhamnose isomerase (L-RI) consistently; avoid switching between L-RI and L-RhI. L-RI catalyzes the isomerization of D-allulose to D-allose, which allows for activity detection via the reduction of the ketose D-allulose in a colorimetric assay based on Seliwanoff’s reaction. Initial optimization was conducted in a single-tube format, where reaction conditions were refined and interfering factors were minimized. This optimized single-tube protocol demonstrated excellent accuracy when validated against high-performance liquid chromatography measurements, confirming its ability to precisely quantify D-allulose depletion. Subsequently, the protocol was successfully adapted to a 96-well plate format, incorporating further optimizations for protein expression and the removal of denatured enzymes. This involved methods such as cell harvest, supernatant removal, and filtration to reduce assay interference. The analytical quality of the established HTS protocol was evaluated using statistical metrics. The results yielded a Z'-factor of 0.449, a signal window (SW) of 5.288, and an assay variability ratio (AVR) of 0.551. All these values meet the acceptance criteria for high-quality of HTS assay. This HTS protocol is highly reliable and applicable for efficient screening of isomerase activity in various industrial and research areas.

## Introduction

Advancements in protein engineering have significantly enhanced biocatalyst capabilities, driven by both structure-based design and empirical data-driven methodologies. Directed evolution (DE), a pivotal technique in this field, mimics natural selection by enhancing enzyme functionalities through increased mutation rates, thereby exploring diverse genetic variations. These innovations have revolutionized biocatalysis, expanding its potential applications across industrial and environmental sectors [1-5].

The extensive libraries generated by DE necessitate efficient high-throughput screening (HTS) for their analysis. HTS enables the rapid screening of large compound libraries, facilitating the identification of specific biological activities or desired functions. This approach allows for the simultaneous, indirect measurement of activities across numerous compounds, obviating the need for individual activity assessments of each variant. Such efficiency significantly complements DE, advancing both the improvement and fundamental understanding of enzymes [6-8].

Target activities in HTS can be identified using a labeled substrate or an indirect sensor system that produces a detectable spectroscopic signal upon reaction. Notably, enzyme activity involving the conversion of reactants or products into sugars can be indirectly quantified through colorimetric analysis. This method utilizes color changes induced by enzyme activity to quantify sugar presence and concentration. Typically, colorimetric sugar analysis involves heating sugar solutions with a strong acid to convert sugar into furfural or its derivatives. Subsequent addition of organic developers, such as indole, orcinol, diphenylamine, or carbazole, results in color generation. Under optimized conditions, these assays provide proportional and consistent results [6, 9].

The visualization of catalytic activity for highly active enzymes through these analyses is crucial for high-throughput analysis of mutant modifications. This is particularly significant in enzyme discovery, enzyme engineering, and drug discovery. Substantial progress in biological sciences is fundamentally driven by the capacity to conduct vast numbers of reactions and screen large volumes of data. This capability deepens our understanding and application of biological processes, further enhancing the scientific community’s ability to innovate and refine biological functions and mechanisms [6, 10].

Monosaccharides, polyhydroxy compounds with multiple chiral carbons, can exist in numerous isomeric forms. While only a few are naturally abundant, the majority are rare sugars found scarcely in nature [11]. Recently, these rare sugars have attracted significant interest due to their low-calorie properties and potential beneficial effects. However, their extraction from natural is resource-intensive, leading to high production costs and environmental concerns. Chemical synthesis is often challenging and can produce toxic by-products, making it unsuitable for food applications. Consequently, the commercial production of rare sugars primarily relies on enzymatic processes. Among the enzymes involved, isomerase is crucial, as it catalyzes the interconversion of ketoses and aldoses, facilitating transformations between different monosaccharides. Thus, isomerase activity is essential for the efficient enzymatic production of rare sugars [12, 13].

This study established a highly efficient isomerase-based HTS protocol, specifically utilizing Seliwanoff 's reaction to detect ketoses, which can act as reactants or products. Enzyme activity was indirectly measured by observing reaction progress and monitoring changes in ketose concentration. Seliwanoff 's reaction distinguishes ketoses from aldoses by dehydrating ketoses in the presence of hydrochloric acid (HCl). Ketohexoses form 5'-hydroxymethyl furfural, while ketopentoses form furfural through dehydration. These compounds react with resorcinol to produce cherry-red or bluish-green compounds, enabling ketose detection [9, 14]. L-Rhamnose isomerase (L-RI) from *Geobacillus* sp., which catalyzes the isomerization of D-allulose to D-allose was employed for HTS protocol. L-RI selected have high-sequence homologous enzyme information was searched in NCBI for L-RhI of *Clostridium stercorarium*, which was previously re-ported as the enzyme affording the highest conversion yield from D-allulose to D-allose [15]. Regarding the base characteristics of L-RI, the optimum temperature and pH were confirmed to be 75°C and pH 7.0. In addition, it showed relatively low substrate specificity for D-glucose and D-fructose, which generally contain a certain amount in the entire process and inhibit enzyme activity as a competitive activity. D-allose, a non-toxic low calorie sweetener, has recently gained attention as a next-generation multifunctional sweetener due to its various functional applications, including anticancer activity and immunosuppressant and antioxidant properties [13]. Ketose substrate D-allulose was monitored to establish HTS protocol using Seliwanoff ’s reaction. Initial optimization was conducted in a bench-top single-tube format, followed by optimization for HTS scale using a 96-well format. The HTS system’s analytical quality was evaluated using statistical metrics to achieve superior performance.

## Materials and Methods

### Experimental Materials

The plasmid harboring the DNA encoding L-rhamnose isomerase from *G*. sp. BMUD, inserted into the pBT7 vector, was commercially synthesized from Bioneer (Republic of Korea). The competent cells *Escherichia coli* BL21 (DE3) and DH5α were purchased from Enzynomics (Republic of Korea). Restriction enzymes (*Eco*RI and *Dpn*I) and *Taq* DNA polymerase were obtained from Enzynomics and Bioneer, respectively. D-allose was purchased from Sigma-Aldrich (USA). Bugbuster Master Mix was purchased from Merck (Republic of Korea). Chemicals for activity assays, including Tris-HCl (pH 7.0, 1 M), Manganese (II) chloride tetra-hydrate, and Resorcinol, were purchased from Enzynomics, Sigma-Aldrich, and Yakuri Pure Chemical Co. Ltd., (Japan), respectively. A 6N-Hydrochloric acid standard solution was obtained from Dae Jung (Republic of Korea). AccuPower Taq PCR PreMix & Master Mix and AccuPrep PCR/Gel Purification Kit were purchased from Bioneer.

### Cell Culture, Expression and Lysis

For individual culture and expression, a single colony from an LB agar containing 50 mg/l ampicillin was inoculated into a 15 ml conical tube containing 3 ml of LB medium supplemented with 50 mg/l ampicillin. Cells were cultured overnight at 37°C with agitation at 200 rpm for 18 h. To induce protein expression, 10 μl of the overnight culture was inoculated into 1 ml of fresh LB medium supplemented with 50 mg/l ampicillin and 5 mM lactose. These inoculated cells were grown at 30°C and 200 rpm for 18 h. Cells were harvested by centrifugation at 13,000 rpm for 3 min, and the supernatant was discarded. Cell pellets were lysed by adding 200 μl of Bugbuster Master Mix and resuspended. The lysed cells were incubated at 25°C for 20 min. Following incubation, the microtube was centrifuged at 13,000 rpm for 3 min to collect the supernatant, which contained the enzyme solution.

For high-throughput analysis, this protein expression protocol was adapted to a 96-well plate format. After harvesting by centrifugation at 2,000 rpm for 10 min, cell pellets in each well were completely resuspended in 200 μl of Bugbuster Master Mix to induce cell lysis. The lysed cells were incubated in a shaking incubator at 25°C and 300 rpm for 20 min. Following incubation, the plate was centrifuged at 4,000 rpm for 20 min to obtain the supernatant containing the enzyme solution.

### Enzyme Reaction Conditions

For individual reactions, 200 μl of the lysate supernatant from the microtube was carefully transferred to a new microtube. Enzyme reactions were initiated by adding 800 μl of substrate master mix, consisting of 100 mM D-allulose, 50 mM Tris-HCl (pH 7.0), 10 mM MnCl_2_, and distilled water. Reactions were incubated in a water bath at 75°C for 4 h. Termination was achieved by heat denaturation at 95°C for 5 min in a heat block, followed by cooling at 4°C for over 15 min in a refrigerator.

For high-throughput analysis, the enzyme reactions were adapted to a 96-well PCR plate format and performed using a thermal cycler (MiniAmp, Thermo Fisher Scientific, USA). Each well received 40 μl of lysate supernatant, to which 160 μl of substrate master mix was added. The thermal cycler was programmed for incubation at 75°C for 4 h, denaturation at 95°C for 5 min, and a final cooling step at 4°C for 15 min.

### Seliwanoff ’s Reaction

The concentration of D-allulose was determined using Seliwanoff ’s reaction [9, 14]. The Seliwanoff 's reagent used in this study was prepared by mixing resorcinol and a 6N hydrochloric acid standard solution. After terminating the enzymatic reaction by cooling at 4°C, the denatured enzyme was removed from the reaction mixture by centrifugation at 13,000 rpm for 3 min. A total of 240 μl of the resulting supernatant was mixed with 480 μl of Seliwanoff 's reagent (1:2 ratio) in a microtube and vortexed thoroughly. The mixture was incubated in a water bath at 60°C for 30 minutes, then cooled at room temperature (25°C) for 1 h to stabilize color development. Following incubation, 200 μl of the reaction mixture was transferred to a 96-well microplate, and the absorbance was measured at 485nm. D-allulose concentrations were calculated using a pre-established standard curve.

For high-throughput analysis, reaction mixtures were filtered through a 0.45 μm hydrophilic PTFE filter plate to remove the denatured enzyme. From each well, 50 μl of the filtered sample was transferred to a 96-well PCR plate, followed by the addition of 100 μl of Seliwanoff ‘s reagent. The plate was briefly centrifuged at 1,000 rpm for 30 sec to ensure mixing. The Seliwanoff ’s reaction was conducted using a thermal cycler programmed for one cycle of 60°C for 30 min and 25°C for 1 h. After the reaction, 100 μl of each sample was transferred to a microplate, and absorbance was measured as described above.

### HPLC Analysis

To validate enzymatic activity and assess the accuracy of the optimized Seliwanoff 's assay, the concentrations of D-allulose and D-allose were quantified by high-performance liquid chromatography (HPLC). The resulting supernatant were filtered through 0.22 μm PTFE syringe filters prior to HPLC analysis. HPLC was performed on an Agilent 1200 Series HPLC system equipped with a refractive index (RI) detector and a Shodex SP 0810 column (300 mm × 8.0 mm). Separation was achieved using distilled water as the mobile phase at a flow rate of 1.0 ml/min. The column temperature was maintained at 80°C, and the RI detector oven was set to 55°C. The concentrations of D-allulose and D-allose were determined from their respective standard curves.

## Results and Discussion

### Verification of the Enzymatic Activity of Recombinant *Geobacillus* sp. L-RI

**Fig. 1:**
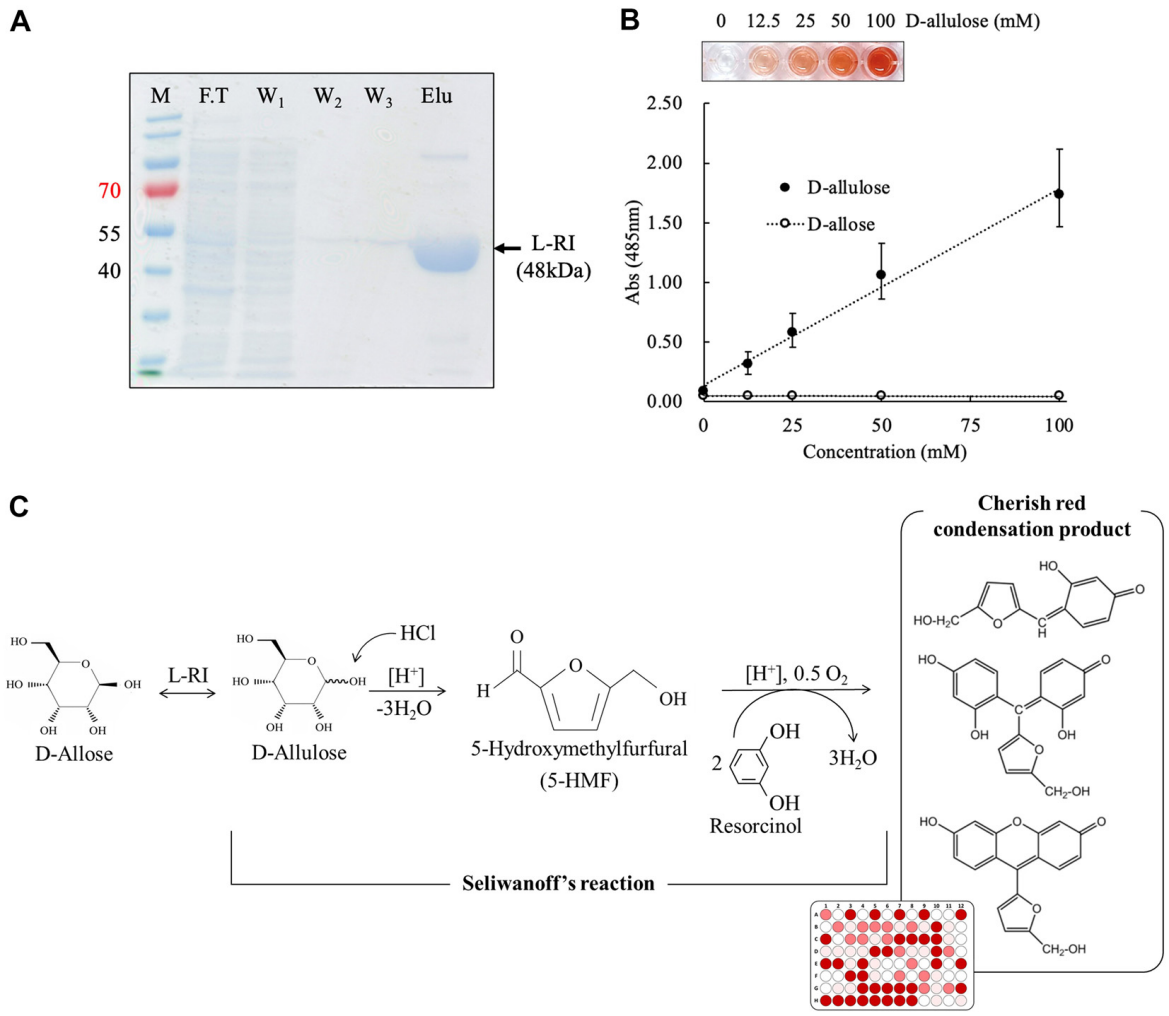
Protein expression and target isomerase reaction (**A**) SDS-PAGE analysis confirming recombinant L-RI expression (M: marker; F.T: flow-through fraction; W: wash fraction, Elu: elution), (**B**) Optimization of Seliwanoff ’s assay conditions using D-allulose standard, (**C**) Schematic overview of the Seliwanoff ’s reaction used in this study to detect D-allulose consumption as an indicator of isomerase activity.

To produce the L-rhamnose isomerase from *Geobacillus* sp., the constructed plasmid was expressed in *E. coli* BL21(DE3) using a lactose induction system. The His-tagged recombinant L-RI was purified from the crude cell extract using Ni-NTA affinity chromatography. The success of the expression and purification was confirmed by sodium dodecyl sulfate-polyacrylamide gel electrophoresis (SDS-PAGE), which showed a distinct protein band at the expected molecular weight, indicating high purity (Fig. 1A).

**Fig. 2:**
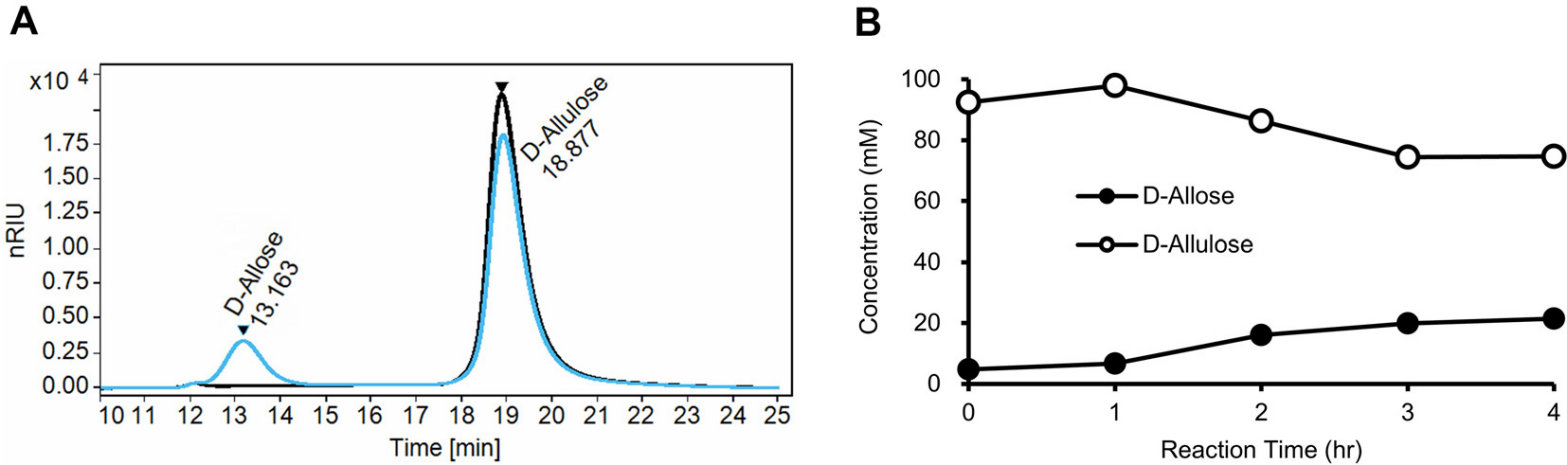
Characterization of L-RI enzymatic activity through HPLC analysis. (**A**) Identification of catalytic activity of purified L-RI by HPLC analysis. The black trace represents the reaction mixture at 0 h (baseline), while the blue trace corresponds to the sample after 4 h of reaction. (**B**) Time-course quantification of the isomerization reaction. Open circle indicates residual D-allulose, and filled circle represents the accumulated D-allose over a 4-h period.

The catalytic activity of the purified L-RI was assessed by its ability to isomerize D-allulose to D-allose. The reaction was conducted at 75°C using 100 mM D-allulose as the substrate and 0.3 mg/ml of purified enzyme. HPLC analysis confirmed the successful conversion; a comparison of chromatograms at 0 h (black line) and 4 h (blue line) clearly showed the emergence of a new peak corresponding to D-allose, concurrent with a decrease in the D-allulose peak (Fig. 2A). A time-course analysis was performed to quantify the production of D-allose. The concentration of D-allose increased progressively, reaching 15.82 mM at 1 h, 21.79 mM at 2 h, and 23.92 mM at 3 h. After 4 h of reaction, the final concentration of D-allose was 24.28 mM (Fig. 2B). This corresponds to a conversion yield of approximately 24.3% from the initial 100 mM D-allulose. The rate of production noticeably slowed after 3 h, suggesting that the isomerization reaction was approaching equilibrium under the tested conditions.

### Optimization of Seliwanoff ’s Assay for HTS Protocol

To facilitate the rapid screening of L-RI variants, HTS protocol was established. The overall process involves cell cultivation and protein expression, followed by cell lysis to obtain the crude enzyme. In the subsequent enzymatic reaction, the activity of L-RI is measured by quantifying the depletion of its ketose substrate, D-allulose, using the Seliwanoff 's reaction. As the critical heating conditions for this reaction were not well-defined for HTS, we first focused on optimizing them. The primary criteria for optimization were achieving a steep, linear absorbance slope corresponding to D-allulose concentration, coupled with minimal standard deviation (SD) across replicate experiments.

The Seliwanoff 's reaction is designed to distinguish ketose from aldoses. That is, the difference in reactivity is due to the structural distinction between D-allulose (ketohexose) and D-allose (aldohexose). This reaction relies on the acid-catalyzed dehydration of D-allulose into 5-hydroxymethylfurfural (5-HMF), which then condenses with resorcinol to form a cherry-red chromophore (Fig. 1C). To determine the ideal reaction conditions, D-allulose standards at concentrations of 0, 12.5, 25, 50, and 100 mM were prepared. Initial trials at 95°C for 5 min resulted in intense signal intensities and showed a linear increase in absorbance at lower concentrations (Fig. 1B). However, at concentrations above 50 mM, the absorbance exceeded 1.0, leading to signal saturation and a loss of linearity, which violates the Beer-Lambert law and compromises measurement accuracy [16].

**Fig. 3:**
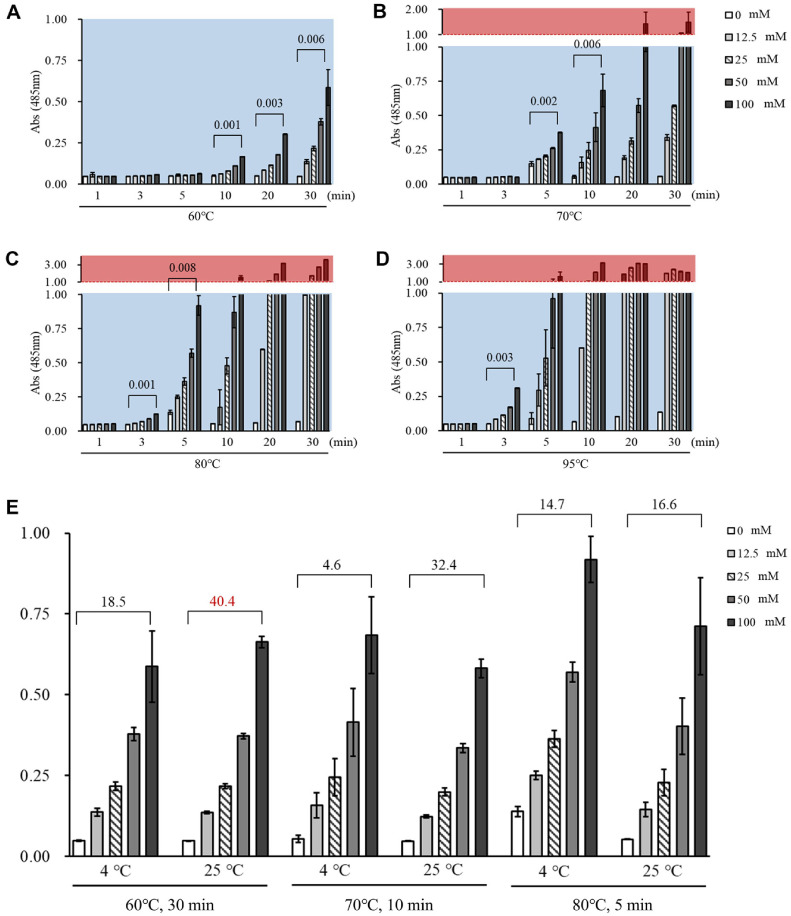
Absorbance of D-allulose at various concentrations (0, 12.5, 25, 50, 100 mM) under different temperature conditions: (**A**) 60°C, (**B**) 70°C, (**C**) 80°C, and (**D**) 95°C. Numbers above each graph indicate the β-values, indicate β-values, representing slopes of the linear regression for each concentration series. Data are shown as mean ± SD (*n* = 3). (**E**) Effect of post-reaction cooling temperature on the absorbance of D-allulose samples in different heating conditions. Numbers above each graph represent the average signal-to-noise ratio (SNR), calculated across all concentration points. Data are shown as mean ± SD (*n* = 6).

To establish a reliable dynamic range, various combinations of lower temperatures (60°C, 70°C, 80°C) and shorter heating times were tested. The optimal conditions were identified as those yielding a steep, linear absorbance slope (R² > 0.99) without signal saturation. The most promising conditions were found to be 60°C for 30 min, 70°C for 10 min, and 80°C for 5 min (Fig. 3A-3C). Conversely, heating at 95°C for 3 min resulted in a low slope (β = 0.003), indicating poor sensitivity, while 5 min led to saturation, confirming its unsuitability for this application (Fig. 3D). In Fig. 3, the shaded rectangular regions are the criteria for optimization. Red rectangle represents the absorbance above 1.0. Conversely, blue rectangle represents the absorbance below 1.0. We optimized absorbance values between 0 and 1.0 to ensure linear accuracy and reliability.

To stabilize the colorimetric reaction and improve reproducibility, a cooling step was introduced after heating. The effect of cooling at 4°C versus standing at room temperature (25°C) was compared. The signal-to-noise ratio (SNR), a measure of assay sensitivity and reliability, was calculated for each condition. The results showed that stabilizing the reaction at 25°C yielded consistently higher SNR values than cooling at 4°C. The combination of heating at 60°C for 30 min followed by stabilization at 25°C for 1 h produced the highest SNR of 40.4 and was thus selected as the final optimized condition (Fig. 3E).

**Fig. 4:**
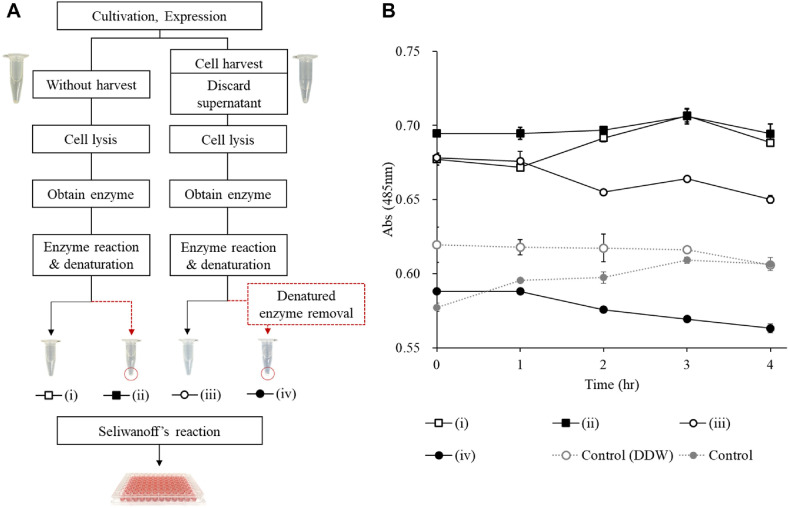
Optimization to minimize assay interference in the HTS protocol. (**A**) Schematic overview of the sample preparation process to reduce interference in Seliwanoff ’s reaction. Samples (i) and (ii) were collected without removing culture supernatants, whereas samples (iii) and (iv) were centrifuged to discard the supernatant prior to reaction. Denatured enzyme removal was performed for samples (ii) and (iv) only. (**B**) Time-dependent changes in absorbance after Seliwanoff ’s reaction for samples (i) to (iv) and controls. Control 1 used distilled water (DDW) instead of the enzyme solution; Control 2 used lysed *E. coli* solution not harboring the L-RI gene as a substitute for the enzyme solution. Data are expressed as mean ± standard deviation (*n* = 3).

With the core reaction conditions optimized, we investigated potential interference from upstream sample components (Fig. 4A). In the L-RI reaction, a decrease in D-allulose should result in a corresponding time-dependent decrease in absorbance. However, reactions using crude cell lysate failed to show the expected decrease in absorbance over time. After removing the denatured enzyme via centrifugation, a clear, time-dependent decrease in D-allulose was observed (Fig. 4B). This result highlights the necessity of a clarification step to eliminate interference from proteins and other cellular components, thereby ensuring the accuracy of the colorimetric measurement. Furthermore, control experiments using distilled water and lysate from *E. coli* not expressing L-RI produced negligible absorbance, confirming the specificity for the D-allulose conversion.

Finally, the optimized Seliwanoff 's assay was further validated against HPLC. An enzymatic reaction was monitored for 10 h, with samples taken every 2 h for parallel analysis. Both methods demonstrated a strong correlation in the depletion of D-allulose over time (Fig. S1A). Although minor discrepancies of approximately 5 mM were observed after 2 h, these were within the SD of replicate experiments. To further assess the level of agreement, a Bland-Altman plot was constructed (Fig. S1B) [17-19]. Most data points fell within the 95%confidence interval, indicating strong agreement and the absence of systematic bias between the two methods [19]. This confirms that the optimized Seliwanoff ’s assay is a valid and reliable method for HTS of L-RI activity. From this validation data, a reaction time of 4 h was selected for future screening, as it provided a statistically significant difference from baseline while remaining practical for HTS.

### Scale-Down and Optimization for the HTS Format

Following the successful optimization of the Seliwanoff 's assay, the entire protocol was scaled down to a 96-well plate format for HTS. A primary challenge in this transition was to ensure uniform conditions for cell cultivation, protein expression, and lysis across all 96 wells to guarantee consistent and comparable reaction outcomes. To maintain sufficient protein expression levels comparable to the single-tube format, an 18-h cultivation and expression period was applied, which was confirmed to yield consistent expression levels across wells via SDS-PAGE analysis. To improve lysis efficiency and reproducibility, incubation at 300 rpm was introduced after treatment with Bugbuster reagent.

The uniformity of this optimized protocol was validated in a two-step process. First, to isolate and confirm the consistency of the physical handling and lysis procedures, a single 100 ml culture was divided into 1 ml aliquots across a 96 deep-well plate. After cell harvesting and lysis with agitation, SDS-PAGE analysis showed highly consistent protein expression patterns, validating the mechanical uniformity of the process. Subsequently, expression and lysis consistency were verified for 96 individual clones, with SDS-PAGE results confirming reproducible protein profiles across the plate (Fig. S2). This result confirmed the suitability of the developed protocol for HTS applications.

**Fig. 5:**
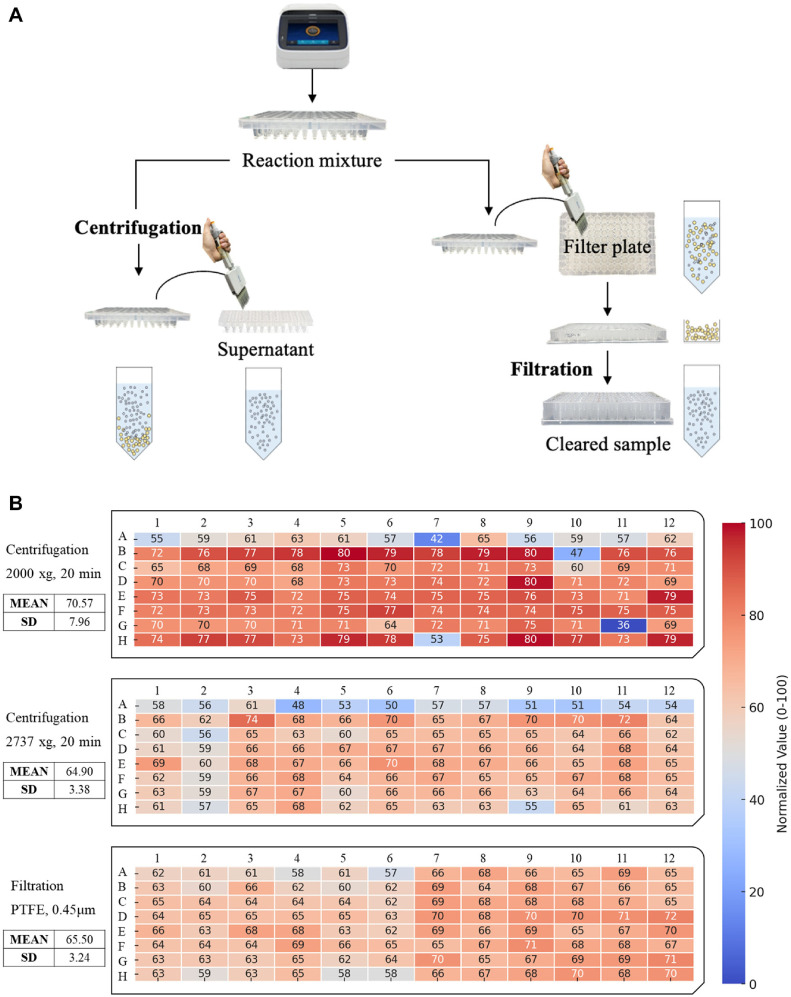
Comparison of denatured enzyme removal methods: centrifugation versus filtration. (**A**) Schematic representation of the workflow comparing centrifugation and filtration-based approaches for removing denatured enzymes in a high-throughput screening format. (**B**) Heatmap visualization of D-allulose concentration in a 96-well plate following isomerization and subsequent Seliwanoff 's reaction under different enzyme removal conditions.

In the HTS format, centrifugation alone was insufficient to effectively remove denatured enzymes from the reaction mixture. Therefore, filtration using a 0.45 μm hydrophilic PTFE filter plate was evaluated as an alternative method for sample clarification (Fig. 5A). After a 4-h enzymatic reaction, Seliwanoff ’s assay was applied to all 96 samples to assess the effectiveness of different clarification methods. Absorbance values were measured pre- and post-reaction, and D-allulose concentrations were determined using a standard calibration curve. To visually assess the consistency of the results across the plate, the final D-allulose concentrations were represented as a heatmap. The heatmap analysis clearly showed that while higher centrifugation speeds improved consistency, filtration with the filter plate provided markedly superior uniformity compared to both centrifugation methods (Fig. 5B). The control data of heatmap is provided in Fig. S3. This finding suggests that filter plates more effectively remove denatured proteins and particulates, thereby minimizing assay interference and enhancing the accuracy and reproducibility of absorbance-based quantification. Therefore, filtration was adopted as the standard method for the HTS protocol to ensure the highest accuracy and reproducibility.

### Quality Assessments of the HTS Protocol

To statistically validate the performance and reliability of the developed HTS protocol, its quality was rigorously evaluated using standard industry metrics. We prioritized the Z'-factor for this assessment, as it is a critical indicator of overall HTS assay quality, reflecting the dynamic range and data variation [20]. In addition, the Signal Window (SW), Assay Variability Ratio (AVR), and Coefficient of Variation (CV) were calculated. Acceptance thresholds for each metric are summarized in supplementary Table 1, with a CV range of 15–20% is generally considered acceptable in biological assays.

Negative controls (enzyme-inactivated at time zero) and positive controls (post-reaction enzyme-substrate mixtures) were used to establish signal baselines for these calculations. As shown in Table 1, all tested conditions met the acceptable CV (%) threshold, confirming low intra-assay variability. However, despite higher centrifugation speeds improving CV (%), SW, and AVR, these values did not meet the acceptance criteria for Z'-factor, indicating insufficient signal separation and high variability. This failure indicates that the centrifugation-based methods suffer from a narrow dynamic range, poor signal separation between positive and negative controls, and high data variability, making them unsuitable for reliable HTS.

In contrast, samples clarified via filtration followed by the optimized Seliwanoff ’s reaction yielded robust performance metrics. The Z'-factor for the filtration-based assay was 0.449, within the acceptable range (0.4–1.0), suggesting good signal-to-noise discrimination. Additionally, SW and AVR values were 5.288 and 0.551, respectively, both satisfying the acceptance criteria. This successful statistical validation confirms that the final HTS protocol, which incorporates filtration for clarification, possesses a good dynamic range, clear signal differentiation, and acceptable consistency. These results provide robust statistical evidence for the reliability and suitability of the developed assay for high-throughput screening applications.

The optimized HTS platform established in this study has great potential for adaptation to other enzyme systems, promising to advance applications in the production of rare sugars and other valuable biochemicals. For instance, this protocol can be directly applied to identify improved L-arabinose isomerases that catalyze the conversion of D-galactose to D-tagatose, a low-calorie functional sweetener, by enabling detection of the ketose product. Furthermore, the principles of this assay may extend beyond isomerases. Through monitoring the depletion of ketose substrates such as D-fructose, the assay can be adapted to evaluate the activity of other enzyme classes, including reductases and kinases, depending on their reaction mechanisms. The versatility and reliability of this robust HTS platform underscore its potential to accelerate biocatalyst development across various biotechnological fields.

## Conclusion

**Fig. 6:**
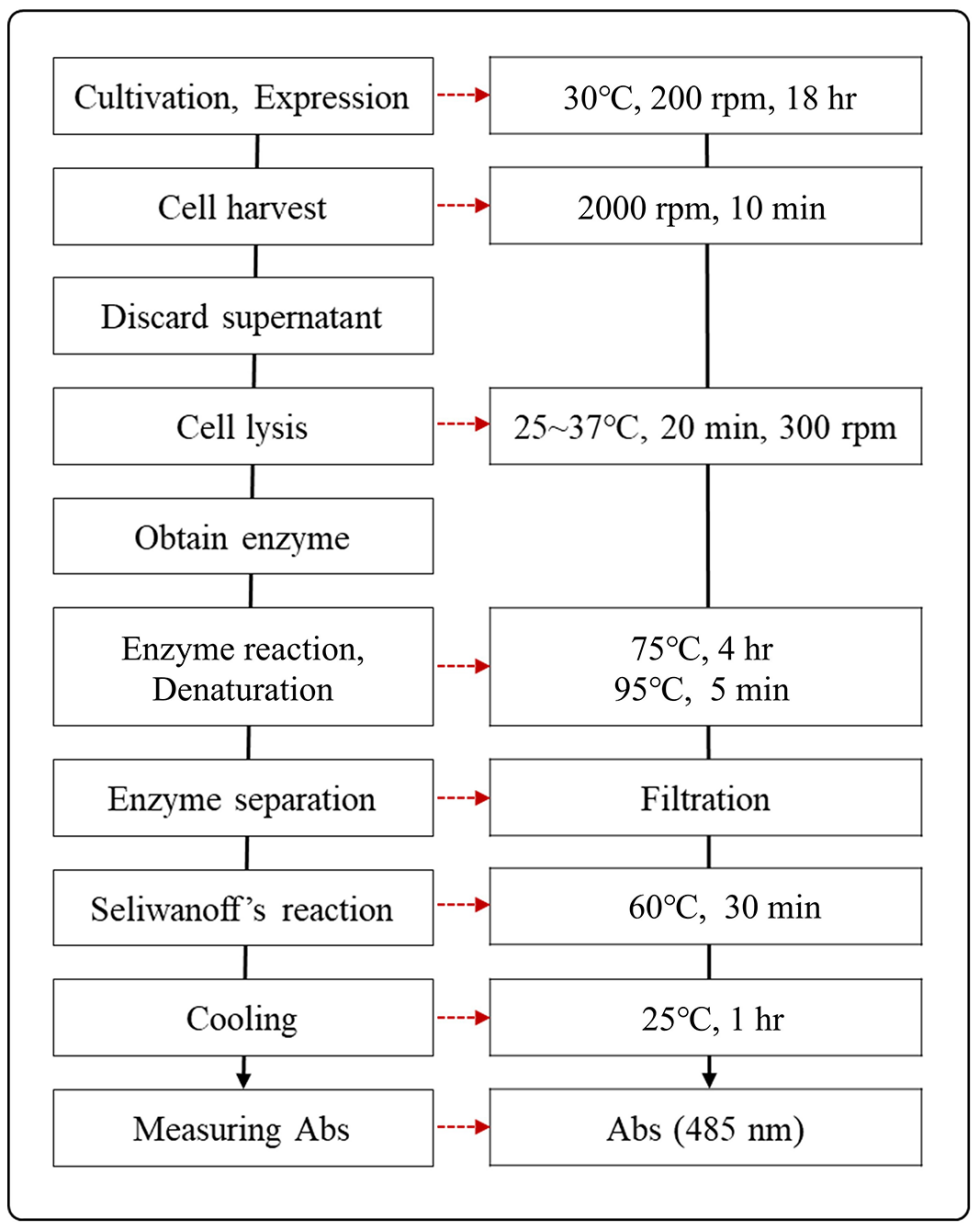
Established high-throughput screening protocol in this study.

In this study, we addressed the need for an efficient method to screen isomerase activity by developing a robust HTS protocol. Using *Geobacillus* sp. L-rhamnose isomerase and D-allulose substrate as a model system, we successfully established a 96-well plate format assay that quantifies enzyme activity by measuring substrate depletion via the Seliwanoff's reaction. The development process involved a systematic, step-by-step optimization, beginning at the bench-top scale and transitioning to the HTS format. This process defined critical parameters for the colorimetric reaction and established uniform conditions for cell expression and lysis. A key finding was that clarification via a filter plate was superior to centrifugation for removing interfering cellular components, which was crucial for ensuring accuracy and reproducibility in the 96-well format. The fully optimized HTS protocol was rigorously evaluated and demonstrated excellent statistical performance (Fig. 6). All quality indicators, including a Z'-factor of 0.449, met the established acceptance criteria, confirming the protocol's high analytical quality and reliability for screening purposes. In conclusion, this work successfully provides a complete and validated HTS workflow for isomerase activity. This system can significantly accelerate the discovery and engineering of novel isomerases, a common bottleneck in industrial biotechnology. The principles established in this study have great potential for adaptation to other enzyme systems, promising to advance applications in the production of rare sugars and other valuable biochemicals.

## Supplemental Materials

Supplementary data for this paper are available on-line only at http://jmb.or.kr.



## Figures and Tables

**Table 1:** Quality assessments of the HTS protocol.

Assay Performance Measurement	CV (%)	Z’	SW	AVR
Acceptance criteria	< 15	0 <	2 <	< 0.6
Centrifugation (2,000 ×*g*, 20 min)	**10**	-0.177	-0.656	1.177
Centrifugation (2,737 ×*g*, 20 min)	**6**	-0.087	-0.4	1.087
Filtration (PTFE, 0.45 μm)	**3**	**0.449**	**5.288**	**0.551**

## References

[1] Bornscheuer UT, Huisman GW, Kazlauskas RJ, Lutz S, Moore JC, Robins K (2012). Engineering the third wave of biocatalysis. Nature.

[2] Currin A, Swainston N, Day PJ, Kell DB (2015). Synthetic biology for the directed evolution of protein biocatalysts: navigating sequence space intelligently. Chem. Soc. Rev..

[3] Arnold FH, Wintrode PL, Miyazaki K, Gershenson A (2001). How enzymes adapt: lessons from directed evolution. Trends Biochem. Sci..

[4] Yang H, Swartz AM, Park HJ, Srivastava P, Ellis-Guardiola K, Upp DM (2018). Evolving artificial metalloenzymes via random mutagenesis. Nat. Chem..

[5] Andorfer MC, Park HJ, Vergara-Coll J, Lewis JC (2016). Directed evolution of RebH for catalyst-controlled halogenation of indole CH bonds. Chem. Sci..

[6] Goddard JP, Reymond JL (2004). Enzyme assays for high-throughput screening. Curr. Opin. Biotechnol..

[7] Buller R, Lutz S, Kazlauskas RJ, Snajdrova R, Moore JC, Bornscheuer UT (2023). From nature to industry: harnessing enzymes for biocatalysis. Science.

[8] Belsare KD, Andorfer MC, Cardenas FS, Chael JR, Park HJ, Lewis JC (2017). A simple combinatorial codon mutagenesis method for targeted protein engineering. Acs Synth. Biol..

[9] Ashwell G (1966). New colorimetric methods of sugar analysis. Methods Enzymol..

[10] Agresti JJ, Antipov E, Abate AR, Ahn K, Rowat AC, Baret JC (2010). Ultrahigh-throughput screening in drop-based microfluidics for directed evolution. Proc. Natl. Acad. Sci. USA.

[11] Granstrom TB, Takata G, Tokuda M, Izumori K (2004). Izumoring: a novel and complete strategy for bioproduction of rare sugars. J. Biosci. Bioeng..

[12] Mu W, Yu L, Zhang W, Zhang T, Jiang B (2015). Isomerases for biotransformation of D-hexoses. Appl. Microbiol. Biotechnol..

[13] Li C, Gao L, Du K, Lin H, Ren Y, Lin J (2020). Production of D-allose from D-fructose using immobilized L-rhamnose isomerase and D-psicose 3-epimerase. Bioprocess Biosyst. Eng..

[14] Sánchez-Viesca FaG, R (2018). Reactivities involved in the Seliwanoff reaction. Mod. Chem..

[15] Seo MJ, Choi JH, Kang SH, Shin KC, Oh DK (2018). Characterization of L-rhamnose isomerase from *Clostridium stercorarium* and its application to the production of D-allose from D-allulose (D-psicose). Biotechnol. Lett..

[16] Swinehart DF (1962). The Beer-Lambert Law. J. Chem. Educ..

[17] Bland JM, Altman DG (1999). Measuring agreement in method comparison studies. Stat. Methods Med. Res..

[18] Bunce C (2009). Correlation, agreement, and Bland-Altman analysis: statistical analysis of method comparison studies. Am. J. Ophthalmol..

[19] Bland JM, Altman DG (2012). Agreed statistics: measurement method comparison. Anesthesiology.

[20] Zhang JH, Chung TD, Oldenburg KR (1999). A simple statistical parameter for use in evaluation and validation of high throughput screening assays. J. Biomol. Screen.

