# Supplementary material for: Establishment of High-Throughput Screening Protocol Based on Isomerase Using Geobacillus sp. L-Rhamnose Isomerase
Source: J Microbiol Biotechnol. 2025 Aug 26;35:e2507026. doi: 10.4014/jmb.2507.07026 (PMC12409428; doi:10.4014/jmb.2507.07026)
Supplement: Supplementary file 1 [file jmb-35-e2507026-supple.pdf]

J. Microbiol. Biotechnol. 2025. 35: e2507026

https://doi.org/10.4014/jmb.2507.07026

Supplementary Figures and Table

Establishment of High-throughput Screening Protocol Based on Isomerase Using *Geobacillus sp*. L-Rhamnose Isomerase

Na Kyeong Koo[1], Sol Min Han[1], Seong-Bo Kim[3], Seung-Ho Baek[4*], and Hyun June Park[1,2*]

[1] Department of Bio-Health Convergence, [2] Department of Biotechnology, Duksung Women's University, Seoul, 01369, Republic of Korea

[3] Bio-Living Engineering Major, Yonsei University, Seoul, 03722, Republic of Korea

[4] Center for Bio-based Chemistry, Korea Research Institute of Chemical Technology (KRICT), Ulsan 44429, Republic of Korea

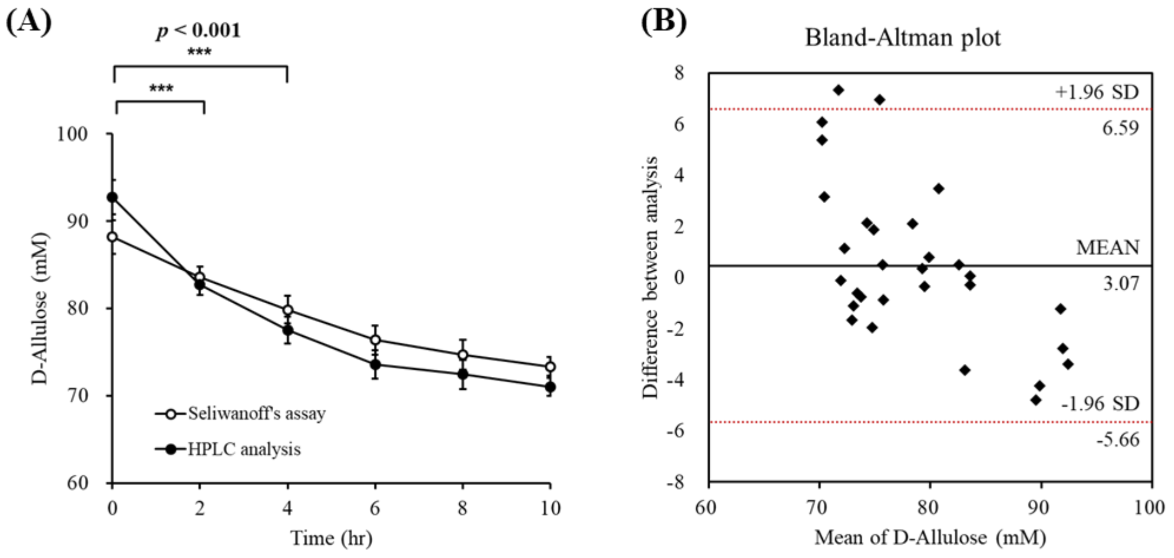

Fig. S1. (A) Comparison of D-allulose consumption during the L-RI reaction as measured by Seliwanoff's assay (open circles) and HPLC analysis (filled circles). **(B)** Bland–Altman plot evaluating agreement between Seliwanoff's assay and HPLC analysis.

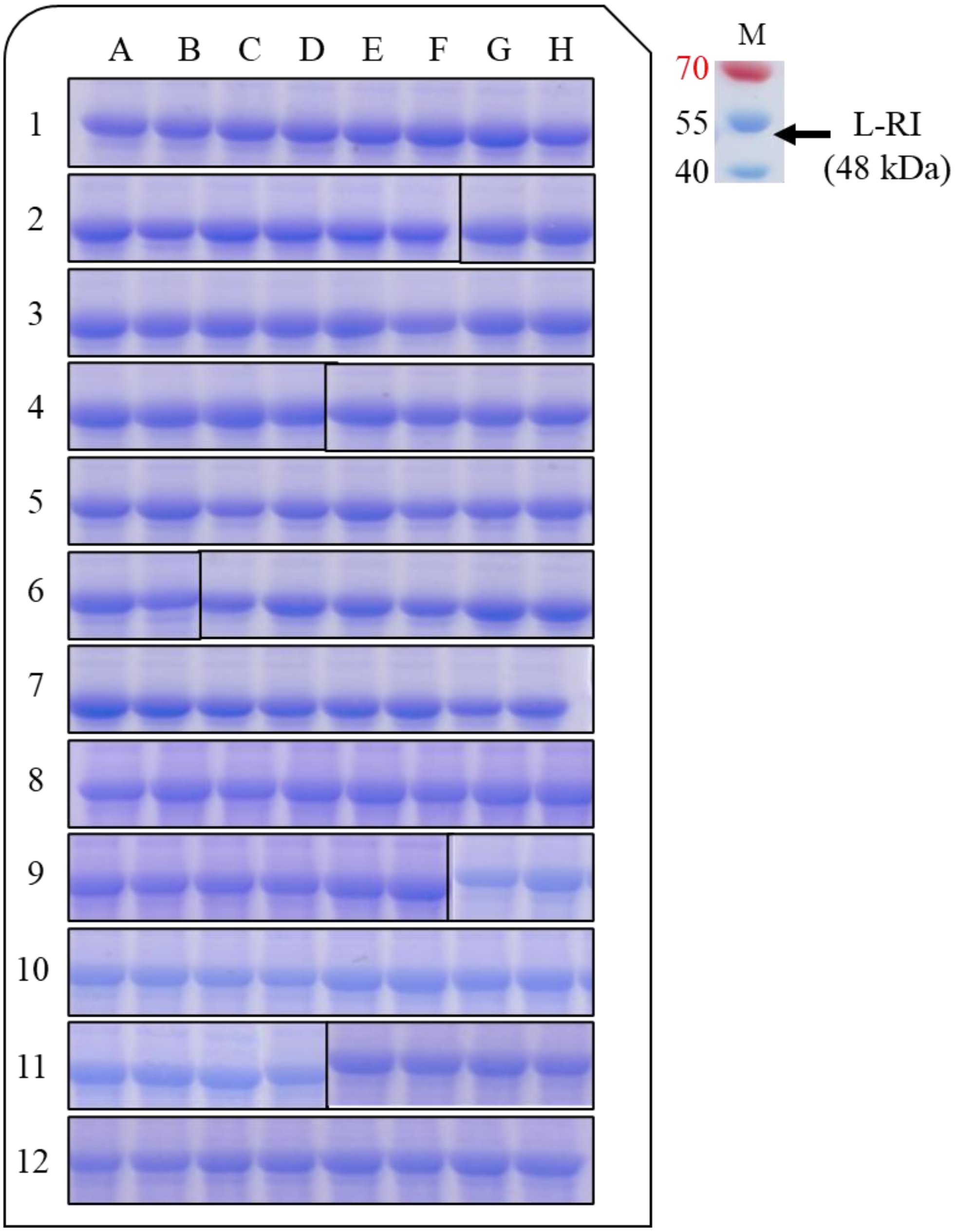

Fig. S2. SDS-PAGE analysis for validation of the mechanical uniformity of the process.

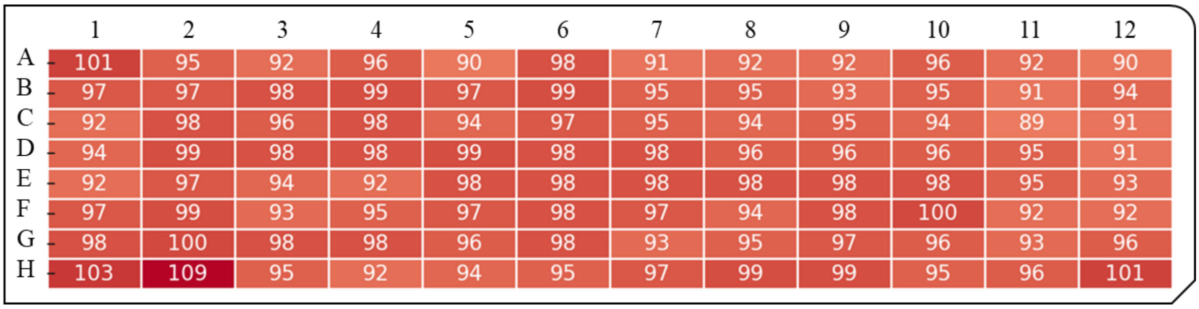

Fig. S3. Heatmap visualization of control reaction of D-allulose concentration in a 96-well plate following isomerization and subsequent Seliwanoff's reaction under different enzyme removal conditions.

Table S1. Acceptance thresholds for the each metric for quality assessment.

Z’	Signal Window (SW)	Assay Variability Ratio (AVR)
Z’ > 0.5 (Excellent)	SW > 2 (Recommended)	AVR < 0.6 (Recommended)
0 < Z’ < 0.5 (Do-able)	SW > 1 (Acceptable)	AVR > 0.6 (Unacceptable)
Z’ = 0 (Yes/No assay)	SW < 1 (Unacceptable)	
Z’ < 0 (Unacceptable)		
